# Role of neuromodulators in regulating Hippocampal encoding and retrieval in anxiety disorders

**DOI:** 10.1186/1471-2202-12-S1-P232

**Published:** 2011-07-18

**Authors:** Ali Hummos, Charles Franklin, Satish Nair

**Affiliations:** 1 Department of Psychiatry, University of Missouri, Columbia, MO 65212, USA; 2 Department of Electrical Engineering, University of Missouri, Columbia, MO 65212, USA

## 

The hippocampus has a critical role in processing context during the acquisition and retrieval of memories. The contextual-dependence of Pavlovian fear extinction is closely associated with the hippocampus [1]. The contextually modulated retrieval of fear extinction leads to limited benefits from exposure therapy for anxiety disorders [1]. We recast extinction learning processing in the hippocampus in terms of a memory task that is dependent on pattern separation and completion machinery in the hippocampus. We developed a spiking neuron model of the Entorhinal cortex, dentate gyrus, and CA3 of the hippocampus. We simulate Acetylcholine and Dopamine effects on regulating pattern separation and completion processes in the hippocampus. Our preliminary results indicate that fear extinction processing can be explained in terms of the encoding and retrieval mechanisms in the hippocampus and accordingly, can be manipulated by neuromodulators. We show that, in our model, manipulating levels of neuromodulators during extinction training can decrease the contextual-dependence of the extinction memory. We conclude that Dopamine is another important modulator of pattern separation and completion in the hippocampus, and that understanding of these neuromodulators will allow targeting specific memories in the hippocampus.

## References

[B1] Bouton ME Context, ambiguity and unlearning: sources of relapse after behavioral extinction Biol Psychiatry 2002 2 976 986 10.1016/S0006-3223(02)01546-9 12437938

